# Green nanobiopolymers for ecological applications: a step towards a sustainable environment

**DOI:** 10.1039/d2ra07707h

**Published:** 2023-04-20

**Authors:** Preeti Chincholikar, Kshitij RB Singh, Arunadevi Natarajan, Rout George Kerry, Jay Singh, Jitendra Malviya, Ravindra Pratap Singh

**Affiliations:** a Department of Chemistry, IES College of Technology Bhopal Madhya Pradesh India; b Department of Chemistry, Banaras Hindu University Varanasi Uttar Pradesh India; c Department of Chemistry, PSGR Krishnammal College for Women Coimbatore Tamil Nadu India; d Department of Biotechnology, Utkal University Bhubaneswar Odisha India; e Department of Life Sciences & Biological Sciences, IES University Bhopal Madhya Pradesh India jitmalviya123@gmail.com; f Department of Biotechnology, Indira Gandhi National Tribal University Amarkantak Madhya Pradesh India ravindra.singh@igntu.ac.in rpsnpl69@gmail.com

## Abstract

To minimize the usage of non-renewable resources and to maintain a sustainable environment, the exploitation of green nanobiopolymers should be enhanced. Biopolymers are generally developed from various microorganisms and plants in the specified condition. This review article discusses the current advances and trends of biopolymers, particularly in the arena of nanotechnology. In addition, discussion on various synthesis steps and structural characterization of green polymer materials like cellulose, chitin, and lignin is also encompassed. This article aims to coordinate the most recent outputs and possible future utilization of nanobiopolymers to the ecosystem with negligible effects by promoting the utilities of polymeric materials like polycaprolactones, starch, and nanocellulose. Additionally, strategic modification of cellulose into nanocellulose *via* rearrangement of the polymeric compound to serve various industrial and medical purposes has also been highlighted in the review. Specifically, the process of nanoencapsulation and its advancements in terms of nutritional aspects was also presented. The potential utility of green nanobiopolymers is one of the best cost-effective alternatives concerning circular economy and thereby helps to maintain sustainability.

## Abstract 2

This perspective presents the role of biodegradable green nanobiopolymers in conserving the environment.

## Graphical abstract

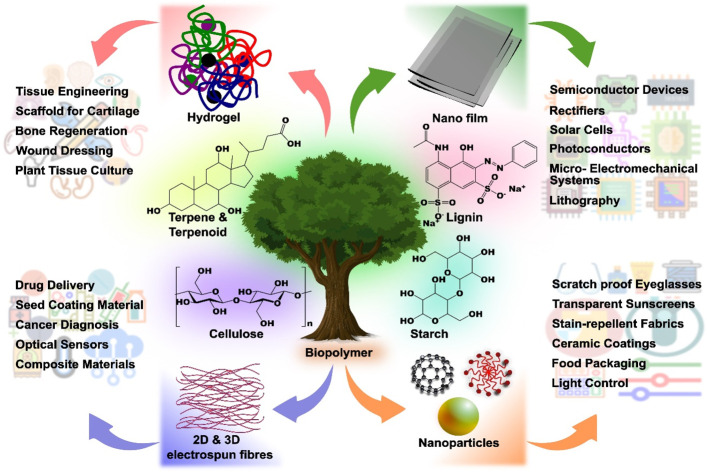

## 1 Introduction

Exhaustive green strategies should be framed to control environmental pollution without affecting the natural resources. Bionanoparticles are low cost, low toxic, and have high mechanical properties, and are preferred for environmental remediation. Blending green chemistry methods with nanotechnology is a holistic approach for attaining sustainability in the future. The bionanocomposites can be used directly or combined with metal oxides to treat hazardous pollutants and salivated water.

The use of polymeric composites is growing widely.^1–3^ These novel polymeric composites have many advantages, like customizable design and requirement-specific modification to sustain the current scientific and industrial demands.^2,3^ Out of all the available polymers, hydrophobic (oil-based) polymers effectively find their way into food packaging materials as they have very little thickness, they are economic, and have extraordinary chemical and mechanical properties.^1–3^ Many hydrophobic polymer base packaging materials are conventionally found and reused in most cases. The more vital assortment of these materials is exploited, leading to environmental pollution.^2,3^ Controlling future concerns due to plastic waste and petroleum sources prompts the formation of materials that are innocuous and of better grade than the biological system materials in present circumstances. Extensive research is being done to synthesize bio-based compounds that can replace polymers extracted from petroleum and allied compounds by exploring eco-friendly products.^1–4^ A distinct class of packaging materials with biocompatible and essential components provides an extra coating of flexible packaging materials and adds to the beneficiary role of the material.^2–5^ Though these polymeric bio-materials are examined for exact use, scientists are trying to address the issues that prevent their widespread use in industry.^3^

Chipping away at these sorts of biopolymer properties can regularly provoke advancements. Low creation levels, contention for food harvests, and higher costs are critical components that could confine the overall usage of biopolymers in packaging advancement. Likewise, experts strive to delimit the mechanical properties and bio-based films. There are limitations to decisions for remaking the bar and packaging equipment structure.^4^ This review targets the recent developments of abundant biopolymers from natural and animal sources, their preparation, their structural characteristics, and, most significantly, their applications. Additionally, scientific limitations and tasks encompassed with bionanopolymers will also be deliberated. Further, it focuses on sources and processes involved in synthesizing biopolymers, like pre-treatment, high-pressure homogenization, and crushing. Followed by this, an elaborate discussion about metal nanocomposites and nanoencapsulation is included. Moreover, it may recommend innovations that advance future scientific research suggestions to mitigate the environmental burden. The nanobiopolymers are promising potential materials due to their featured characteristics like abundance, biodegradability, and non-toxicity. The main purpose of this article is to inculcate budding researchers to preserve the environment using biodegradable compounds.

## 2 Bionanopolymers

Biopolymers are chains of biomolecules composed of repeating monomers, mainly covalently joined.^1,2^ A collection of materials, mostly made from natural resources to delineate organisms, crops, or even plants, is characterized by the articulation “biopolymer. “Materials-based particles created from natural resources such as vegetable oils, carbohydrates, lipids, saps, proteins, amino acids, and so on are known as biopolymers.^2–4^ In contrast to conventional polymers, which have a less complex and irregular arrangement, biopolymers are complex sub-atomic collections with 3D engineering frameworks.^5^ It is unquestionably essential to provide biopolymers with dynamic atoms *in vivo*. Their position relies heavily on their predefined shapes and organization. Biopolymers are categorized in various ways, as indicated by different scales.^5,6^ Based on the functional groups in the polymer chain, biopolymers are classified as polycarbonates, polysaccharides, polyamides, polyesters, and polyvinyls. These classes are additionally characterized according to their source into various sub-categories.^3,4^ Based on the repeating unit, biopolymers are divided into three classes: (i) polysaccharides, which are sugars, (ii) proteins, which are amino acids; and (iii) nucleic acids, which are nucleotides. Biopolymers are well-known for their roles as bioplastics, biosurfactants, biocleansers, bioadhesives, bioflocculants, *etc.*^6,7^

### 2.1 Sources and synthesis of biopolymer

Biopolymers are plastic derived from environmentally friendly biological sources.^6,7^ The presence of biopolymers in plants suggests a bioeconomic prospect for their position. Typical artificial polymers are mass-produced before being crafted with the final objective of coherent assessment efforts. Microorganisms play a significant role in forming biopolymers such as polyesters, polysaccharides, and polyamides. The polymer's composition was influenced by its proper attributions, number of repeated units, and sub-nuclear heap.^8^ The physical and chemical properties of a collection of biopolymers fused with the assistance of living beings may be individually tailored to the consistent therapy of microorganisms, making it appropriate for clinical applications such as medication movement and tissue instrumentation.^9^ Biopolymers produced by microbes require immediate nourishment and constant monitoring of their surroundings. These are mechanically progressed by direct development or compound polymerization by employing repetitive units appropriately produced *via* maturing. Most biopolymers are biocompatible and biodegradable without adverse effects on natural systems. The practical approach for assembling biopolymers is typically considered due to their specific resistance or as the limit material.^9^ It has been shown that these materials are frequently contaminated by natural cycles, allowing them to be reabsorbed in the environment.^9,10^ By concentrating our attention, to a greater extent, on biopolymers, we can accomplish a significant reduction in CO_2_ toxins while maintaining gratifying progress.^10^ Green development is an excellent feedstock for the plastic age due to its high production and flexibility.^9,10^ Green advancement demonstrates the possibility of utilizing carbon and destroying ozone-harming chemicals discharged from many current industries. Green development-based plastics have been a recently joined inclination in bioplastics in connection to conventional methods of employing corn and potato feedstocks as plastics.^1,10^ Even though green development-based polymers are still in the preliminary stages, they will find uses in a broad range of activities once commercialized. At the moment, microbial plastics are regarded as a vital substance of polymeric material with promising commercialization. They can modify fluid enhancement parameters, exemplify materials, flocculate particles, and produce emulsions and suspensions.^10^

## 3 Green polymer materials and their structural properties

Biopolymers find many applications in numerous fields and are the most desirable biomaterials. Due to the polymer's design and surface features, the biomaterials' unique characteristics may regulate diverse functions.^9,10^ Polymer-based materials formed from natural resources assist in producing functional composites through economically viable and biologically safe fuels.^1,9^ A green polymer entity accepts a composite material with a polymer/polymeric compound/biodegradable material obtained unambiguously from a reliable source. That, in return, provides a wide range of polymers with conventional biodegradability features and will replace regular oil-based polymers.^1–3^ Proteins, starch, chitin, and cellulose are typical examples of polymers.^1–3^ Furthermore, alternative green polymers include regular flexible plastic (NR), corn-gathered polylactic destructive (PLA), and bacterial production of polyhydroxyalkanoates (PHA). In general, polymers such as poly(α-hydroxy butyrate), poly(ε-caprolactone), poly(vinyl alcohol), poly(methyl methacrylate), poly(dimethylsiloxane), PU, cellulose, silk, and others are employed for varied applications.^2,3^

### 3.1 Cellulose

Cellulose is obtained from abundant biopolymers and stable sources.^2,10^ Downy, hemp, and cotton are examples of solid cellulose. Sugar belongs to the polysaccharide^11^ arrangement because it comprises monomeric units of cellulose. Normal cellulose (C_6_H_10_O_5_) introduces polysaccharides, in which hundreds to thousands of 1–4 linked d-glucose units immediately solidify.^10–13^ Generally, plants have a usual cellulose content of 33%.^12,13^ Cotton and wood are among the plant-based arrangement that is rich in cellulose. Cotton contains 90% cellulose, while wood has half or more cellulose content than cotton.^12,13^ Despite cellulose, plant fibers contain specific parts like lignin, hemicellulose, and gelatin. Since cellulose is an abundant fundamental element in nature and has charming properties, it is considered to satisfy the need for an eco-friendly polymer with bioavailability.^12,13^ Cellulose is insoluble in several solvents, limiting its activation and use.^11–13^ Recently, cellulose strands have been considered a significant assist in thermoplastic polymer groupings. This is due to properties of cellulose like low thickness, minimal wear resistance, a separated functionalized surface, inexpensive nature, and vital resistivity.^13^ Compared to polymer grids stacked with inorganic fillers, cellulose-derived polymer composites may be readily terminated during the reuse framework.^13,14^ Despite these various advantages, the use of cellulose fibre on a large scale is limited. This problem is explained by the inability to get excellent spread values in the polymer lattice.^14^ It is important to note that cellulose is a polysaccharide that contains glucose as its monomeric unit. Fructose and galactose are present in primary glucose isomers.^14,15^

Glucose particles are connected throughout the cellulose chain by glycosidic linkages, caused by a lack of hydrogen atoms beginning with one monomer and continuing onto the following and hydroxyl bundles in another monomeric unit.^15^ It will promote the formation of microfibrils.^14^ Intermolecular hydrogen bonds hold together these microfibrils as they approach fibrils. In cellulose homopolymer, d-anhydrous glucopyranose units are restricted by (1–4) glycosidic connections. Glucose is called pyranose because it is a six-membered ring in cellulose structure.^15^ The phrase “basal cellulose” refers to cellulose in two forms of glass-like design, cellulose I and cellulose II. The biopolymer cellulose II is formed when cellulose I is exposed to sodium hydroxide. There are more cellulose structures outside type I and type II, such as cellulose III and IV. Cellulose I is less persistent than other polymorphisms, but cellulose II is the most difficult to produce.^16^

Polymorphism is a property by which the compound is accessible in multiple structures. Cellulose contains different hydroxyl bonds, intramolecular and intermolecular hydrogen bonds, provoking other coordinated arrangements.^16,17^ The cellulose unit has six hydroxyl groups and three oxygen atoms. There will now be many access points for modifications in the ring, different cellulose units, and chain length. Cellulose polymorphs are grouped into four types in general.^17^

**Fig. 1:**
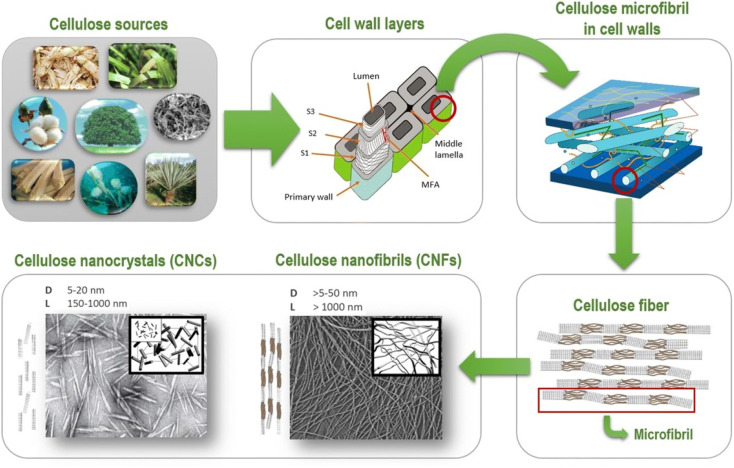
Schematic representation of cellulosic fibres (Reproduced from ref. 19 with permission from Springer, copyright 2020).

Cellulose I is more abundant in the environment.^2,3^ Atala and Vanderhart, 1984, used NMR to describe cellulose and exposed that it exists in two forms, Iα and Iβ.^17,18^ The molecule comprises a triclinic unit with a hydrogen limiting chain for each unit cell, with similar van der Waal associations to cellulose.^17,18^ Each unit cell comprises two hydrogen limiting chains and a monoclinic unit. These two forms of cellulose coexist, and their rates alter depending on the kind of cellulose.^18^ Cellulose obtained from crude organic entities (microbes, green growth) has high cellulose Iα content, whereas cellulose obtained from plants has low cellulose Iβ content. Modifying cellulose Iα in particular solvents at 2600 °C to 2800 °C results in a more stable cellulose Iβ.^18^ The nanocellulose can be obtained in various forms and shapes, namely cellulose nanofibrils I (CNFs) and cellulose nanocrystals II (CNCs), shown in Fig. 1.

Cellulose II is more thermodynamically stable than cellulose I. John Mercer designed the treatment procedure in 1844 to convert cellulose I to II.^17,18,20^ The cellulose is treated with a soluble base during the interaction at convergences of approximately 17–20% w/v.^17,18,20^ It prompts irritation of the cellulose fiber when it shapes a space between the cellulose atoms with no separation. During the interaction, cellulose atoms' equivalent chain path is changed into equivalent reverse chains. Another kind of degradation and recovery is the conversion of cellulose I to cellulose II.^18,20^ As the name indicates, this cycle includes the complete breakdown of cellulose and the recovery of cellulose strands. Several processes can retrieve cellulose, including copper ammonium and *N*-methyl morpholine *N*-oxide.^20^ Phosphoric acidic recovery has been used to account for the cellulose I to II transitions. The irreversible linkage between cellulose I and II indicates that cellulose II is more thermodynamically stable.^20^

Cellulose III is found in two forms: aromatic salt of cellulose I yield cellulose III, and antacid produces cellulose III. Yatz immersed cellulose in a salts scheme in 1986 by converting it to cellulose III and then degassing it.^21^ The conversion to cellulose III is reversible, where the chain direction does not alter. Cellulose IV is formed during reverse change by treating cellulose III with glycerol at high temperatures. Hermans and Weidinger employed cellulose units in 1946 with the option of converting cellulose strands to cellulose IV by treating them at high temperatures with glycerol.^22^

#### 3.1.1 Nanocellulose

One of the essential biodegradable and stable microcomponents found in nature is nanocellulose. Nanocellulose is fundamentally concerned with cellulose on a nanoscale scale^21–23^ and has produced cellulose from wood, plants, waste, and plant materials since the 1970s.^23,24^ Turbach, Snyder, and Sandberg effectively organized smaller-than-usual fibrillated cellulose in 1970.

Whenever the plant cell divider exists, it produces some disturbing effect; consequently, cellulose fibers changes. Hence, the strands become nanofibrils (CNFs) or their microfibril assemblies (CMFs), which range from 10 to 100 nm depending upon their condensing point.^25–29^ Standard processes like homogenization, microfluidization, squashing, cryo crushing, and ultrasonication will produce good-quality cellulose nanofibrils and cellulose microfibrils. Nanocellulose assembly entails several processes, including mechanical and chemical treatments. These drugs enhance the distinct stable linking of microfibrils found in normal cellulose.^24,28^ The exact phrases and the length of the phase define the kind and quality of the final nanocellulose product. The duration of de-coordinating beyond the crude material determines the extent of the final nanocellulose.^25,30^ There are at least one or two extraction processes for the occurrence of nanofibrils. They are regularly completed by mechanical means, for example, smashing liquid nitrogen, cryo-crushing, high-pressure homogenization, *etc.* Unique solvent bases and enzymatic hydrolysis are used to nurture the advantage of hydroxyl dynamic linkage before mechanical systems work on the inner surface, change crystallization, and decrease cellulose hydrogen bonds.

#### 3.1.2 Pre-treatment for nanocellulose formation

**Fig. 2:**
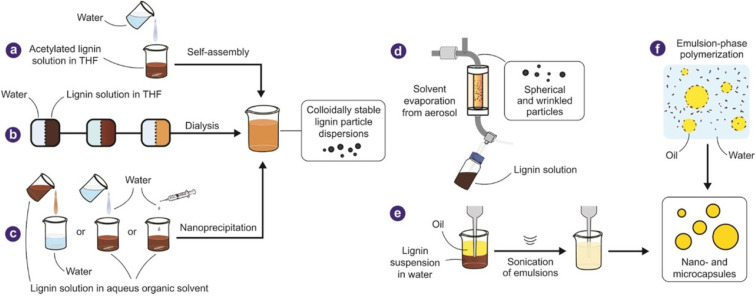
Methods of fabrication of lignin (Reproduced from ref. 33 with permission from John Wiley and Sons, copyright 2019).

Two significant concerns often occur during the fibrillation stage, particularly during mechanical fibrillation of cellulose, are (i) fibril, when the slurry is drained off *via* the softening device, and (ii) fibre connection.^28–31^ The suspension might contain different feeds until the prevailing exfoliate of the cell dividers happens. The motivation behind delivering nanofibers requires massive energy assurance. In light of past logical investigations to reduce interfibrillar hydrogen bonding, viable pre-treatment decreases energy utilization. The pre-treatment strategies rely upon the cellulose's source.^31^ Proper pre-treatment of cellulose filaments upholds quality, works on the inner surface, changes crystallization, lessens energy concentration, and advances nanocellulose formation.^32^ For instance, pre-treatment of vegetables, harvests, organic products, and established materials can build the aggregate or restricted expulsion of non-cellulose parts, for example, hemicellulose, lignin, and the detachment of explicit filaments. Tunicate's pre-treatment incorporates the partition of particular cellulose strands, the expulsion of the protein network, and the division of layers.^28–32^ Pre-treatment of green growth ordinarily assists with eliminating the framework material of the green growth cell dividers; however, pre-treatment of bacterial NCs is pointed toward eliminating microorganisms and foreign substances from the arrangement.^31^ Pre-treatment is essential since it incorporates the entire formation, crystallinity, and variety of the cellulose and many of the highlights of the previously treated raw material. Cell exfoliation is a pre-treatment technique that aids in forming nano-sized strands. Pulping tactics, dying, primary corrosive soluble treatment, enzymatic treatment, ionic fluids, oxidation, and steam impact are unique pre-treatment approaches.^1,30–32^ The fabrication technique used to synthesize nano and micro-level lignin is shown in Fig. 2.

#### 3.1.3 High-pressure homogenization

High-pressure homogenization is one of the significant innovation technique used for the lab-level creation of nanofibrils^34,35^ and CNFs. Much of this cycle includes pushing the arrangement utilizing a circle and a cylinder at raised pressures of 50–2000 MPa,^34,35^ or another bulky network. The hole width relies upon the applied strain and the thickness of the arrangement.^35^ The subsequent high-performance streaming speed diminishes the unique strain force and the steady tension under the fluid stage. It contributes to the formation of gas bubbles, which rupture as the fluid exits the homogeneity hole under normal air tension.^34,35^ This improves the development of gas bubbles, which rupture again when the fluid leaves the homogeneity hole under standard air tension.^34,35^ Gas bubble creation and the rupture can create effects and depressions, setting off influences in the arrangement of cellulose. Critical pressure decrease, high shear powers, and interparticle impact accomplish a deterioration in cellulose fiber size. The degree of cellulose is still in the air concerning the scope of homogenization cycles and the increased strain.^34,35^

#### 3.1.4 Microfluidizer for micro liquefaction

Microfluidizer is an additional advancement used to make cellulose nanofibrils or cellulose microfibrils. The microfluidizer operates at a consistent frequency, whereas a homogenizer works at a reliable strain. The liquid-inclined design is routed through a *z*-shaped chamber with a shear force of ref. 35 and 36. The strain may be detected at a limit of 40 000 psi, approximately 276 MPa.^35^ Pre-selected microchannels are deliberately constructed inside the chamber, and the activity course is zoomed to high speeds. When the slurry stream comes into contact with wear-safe surfaces, it creates a critical impact force. Different check valves engage slurry dispersion. After leaving the association portion, it is transported to a hot exchanger. It is then re-streamed into the system for additional movement or sent according to an outer point during the accompanying action. The procedure should be repeated several times to allow various examined chambers to assemble the amount of fibrillation. The course of action in the microfluidizer affects the morphology of the extracted cellulose nanofibrils.^37^ They observed that the point extension of nano fibrillar packs rises after 10–15 trade cycles; however, excessive movement induced CNF buildup due to the lengthy surface region and the significant number of surface hydroxyl bundles.^38^

#### 3.1.5 Crushing

The distressing framework is another procedure for separating cellulose strands into nano-sized fibers.^39^ During the distressing framework, the fiber fibrillation movement is ended by moving cellulose between a fixed and a turning grindstone at about 1500 rpm, which gives shear strain to the strands.^39^ The fibrillation association in the processor separates the cell division arrangement and the nanoscale strands.^38,39^ The distance between the plates, the nature of the circular tunnels, and the feed count in the processor influence the fibrillation intensity. A couple of runs through the homogenizer have been recommended to produce fibrillated cellulose. Replacing and staying aware of circle stones is counterproductive, as wood crush strands can dissolve coarseness close to scratches.^39,40^ Regardless, the critical advantage of processor movement is that it generally doesn't require additional mechanical pre-treatment.^40^

#### 3.1.6 Pounding

Cryocrushing is an excellent mechanical fibrillation technique for cellulose. This cycle generates strands with extensive lengths that change between 0.1 to 1 m. Water-stirred cellulose fibres are typically chilled in liquid nitrogen before being uniformly crushed.^39,41^ Because of the pressure supplied by the ice stones, applying significant percussion abilities to the frozen cellulose strands contributes to the dissolution of the cell dividers. Cryo-crushed fibres can be diffused and fairly disseminated in water using apparent putrefaction.^42^ This method relates to various cellulose materials, with the fibre pre-treatment procedure before homogenization. The makers made nanofibers from soybean stock through high-pressure fibrillation and cryo-crushing. TEM has emphasized that nanofiber widths range from 50 to 100 nm.^41^ The nanofibers show excellent dispersion in acrylic emulsions than in water. Regardless, cryo-crushing development offers a low capacity and high energy cost.^42^

#### 3.1.7 High-intensity ultrasonication

Intense center ultrasonication is the most broadly perceived lab mechanical therapy procedure for liquid conditions^43,44^ for cell agitating impacts. This advancement produces possible cavitation, like the chance of trials, expansion, and the breakdown of small gas ascends after water particles control ultrasonic energy. The accomplishment of ultrasound's hydrodynamic abilities contributes to cellulose strand defibrillation.^43^ Several researchers have examined the efficiency of focused energy ultrasonication (HIUS) for separation nanofibers from various cellulosic materials, like plain cellulose, microcrystalline cellulose, crushed, fried banana strip, rice trash, and micro fibrillated cellulose.^3,17,43,44^ The results show that fast mixing of small and nanoscale strands can be achieved after ultrasonication of cellulose. The distances across the eliminated fibres range from 20 nm to a couple of microns, indicating that some nanofibrils rise from the fibre.

In contrast, others remain on the fiber's external layer.^43,44^ As a result, it provides massive width transfer to linked fibres. It can also be noted that the ultrasonic treatment approach has altered the apparent growth of some cellulose fibres.^43^ These changes depend on the type of cellulose sources; for example, the crystallinity of cellulose increases for 100 percent pure cellulose. Using HIUS treatment, the researchers investigated the effects of distance from the test tip on temperature, fixation, strength, adequacy, length, and the degree of fibrillation of several cellulose strands.^44^ They found that the long strands are astoundingly low defibrillators and are the best fibers due to their high strength and temperature. The high squash content and the massive separation from the test to the estimating utensil are not helpful for fibrosis.^44^ A blending of HIUS and HPH extends the homogeneity of nanofibers, but fibrillation appears differently. The oxidized squash is used for HIUS treatment,^43,44^ thereby improving the development of nanofibrils cellulose.

#### 3.1.8 Destructive hydrolysis

To eliminate cellulose nanocrystals, destructive hydrolysis of pure cellulose material can be done under-regulated environment, time, and inert environment.^45^ For this explanation, mineral acids such as sulfuric acid, hydrochloric, phosphoric, malic, hydrobromic, nitric, and formic acid are employed.^3,17,45^ Sulfuric acid is the most often used for forming cellulose nanocrystals. During hydrolysis, unclear sections of cellulose and interfibrillar connections are hydrolyzed without disturbing the glassy nature of the nanocrystalline material.^45^ The cellulose nanocrystal dispersal in intense destructive is cleaned using water and by perpetual centrifugation.

The nanocellulose can also be prepared from synthetic pre-treatment processes like oxidative, enzymatic, and acid hydrolysis.

### 3.2 Chitin

Chitin is a characteristic polymer commonly found in shells and fish scales. Chitin was shown to be the most prevalent polymer in nature after cellulose. Chitin and cellulose are both members of the polysaccharide class. Chitin differs from cellulose because it has an acetamide derivative rather than a hydroxyl group. Crab and shrimp shell garbage from the fishing industry contains 8–33 percent chitin polymer. Chitin comprises monomer units of B-1,4-*N*-acetyl-d-glucosamine that are aligned in a straight line, as seen in ref. 46 and 47. The selection of shells is the first step in the separation of chitin. In an ideal world, shells of comparable size are chosen. Tolerably thin dividers make chitin recovery more useful for shrimp shells.^46,47^ The subsequent stage in the technique is washing and drying the shells after extensive crushing. A small portion of shell fragments is eliminated with dilute hydrochloric acid to remove calcium carbonate. The soluble base treatment removes the protein, surrounded by other typical contaminations (20% sodium hydroxide). The darkening, characteristic carotenoids are extracted using ethanol in a disintegration technique.^47^ Chitin has biodegradability as well as antimicrobial properties. Chitosan is insoluble in water and impervious to soluble bases, acids, and various solvents.^46–49^ For the most part, chitin is used in various applications, for instance, biosensors and prescriptions. In the clinical field, chitin is used as a wound dressing material. Chitin, a bioavailable, polyhydroxy polymer with good optical properties, is used for coating metal particles.^46,49^

Chitin does not provide a hazy or scattered illustration. Chitin and cellulose have outstanding characteristics. Chitin, like chitosan, is not a water-soluble polymer but is easily dissolved in common solvents. Chitin is soluble in hexafluoroacetone, hexafluoro isopropanol, and chloro alcohols, including mineral acids.^46,47,50^ Chitin begins to deteriorate before condensing due to the presence of hydrogen bonding. In the microfibrillar structure of chitin, there is more possibility fora protein network. The microfibrils' size was measured in the 2.5–2.8 nm range. Chitin is thixotropic and has a liquid glass-like structure in the crab shell. Various researchers have detected the glass-like arrangement of chitin from squid pens and crab shells.^47,50^ Destructive hydrolysis is done using hydrochloric acid to disengage the distinct regions of chitin. It has been accounted that the chitin chooses its stimulating edge in the polymer structure.

The development of chitin and chitosan has been investigated in many research articles.^50,51^ The precise kinds of chitin, namely α-, β-chitin, depends upon the polymer chains' arrangement and course of action.^51^ In their study of the ring structure, the researchers discovered that A-chitin and B-chitin derived from shrimp shells and squid pens exhibit relative diffractograms in XRD analysis.^52^ XRD data of considerably transparent models confirmed the glass-like growth of a- and b-chitin.^52^ However, the glass-like boundaries for the two types of chitins are exceptional. Each a-chitin unit cell contains two reverse particles.^52^ Alternately, b-chitin has been represented to have equivalent strategies per unit cell. Regardless of such differentiations, the two kinds of chitin show a free clear unit for the *N*-acetyl glycosyl utilitarian social occasion.^52^ Various systems are available for the fundamental justification behind investigating the physico-chemical properties of chitin and chitosan.

XRD models for a-chitin obtained from various sources, such as lobster and sagitta, revealed differences.^52,53^ The diffraction at the 110 plane was found on the exclusive cross-segment due to a-chitin obtained from lobster; at this point, no comparable a-chitin was obtained from Sagitta^51^ at an equivalent peak position. a-chitin from Sagittarius has been considered more glass-like and appeared differently concerning a-chitin obtained from lobster.^51^ Thus, much thought ought to be paid to investigating the development of a-chitin to decide ambiguities.^51^ Nevertheless, the undeniable beneficial stone plan for b-chitin is especially filed rather than specific issues associated with unit cell limits. The precise arrangements of commercial chitin and chitin removed from the blended beverage protein treatment procedure were 97.9 and 81.0%, independently (design 2*θ* = 16). Overall, it was seen that the crystallinity of chitin in enzymatic treatment using Protease blended drink^53^ was reduced from 97.9% to 88.0% in commercial chitin. The degree of acetylation and crystallinity index plays a significant role in determining the physical parameters of chitin, protease chitin, alkali, and acid treatment chitin.

### 3.3 Lignin

Lignin is the second most abundant biopolymer on earth after cellulose. It possess promising high-level properties, like high antimicrobial activity, cancer prevention agent, low thickness, excellent hardness, high carbon content, and photometric properties.^51–54^ With such qualities, a broad examination proposes changing over lignin into eco-friendly products. Lignocellulosic materials contains cellulose, lignin, hemicellulose, small amounts of wax, and water-solvent mixtures. In standard filaments, the material's strength depends on nature of cellulose, warm dampness assimilation, and biodegradation. Despite its excellent properties, lignin is responsible for the biodegradation of UV light.^54^ Because it is hydrophobic, it prevents water from entering the phone panel and ensures adequate water and supplement transmission in the phones. Because of the phenylpropanoid monomer structure, lignin is a cross-connected macromolecular substance.^53,54^ The subatomic mass of lignin is estimated to range between 1000 and 20 000 g mol^−1^ when separated from diverse sources. Lignin has a broad range of randomly arduous designs. It frequently separates during extraction, making the polymerization rate extremely difficult to assess, for example, in high crosslinking polymers. The interaction and the source of lignin extraction, such as hardwood/softwood/grass, determine the characteristics of lignin.^53,54^

**Fig. 3:**
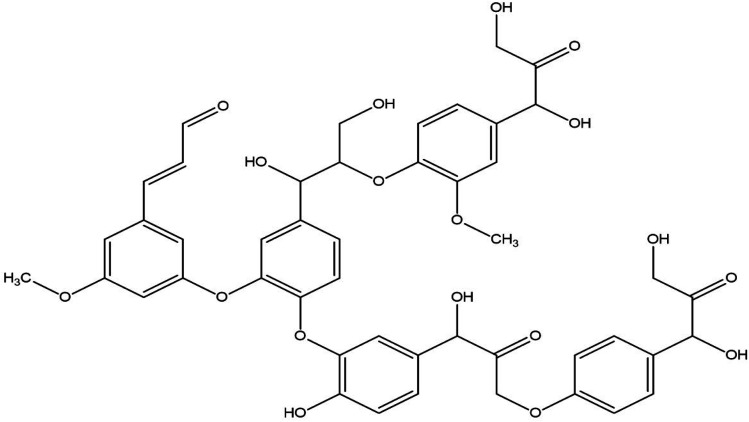
Structure of Starch.

Watkins *et al.* showed that the lignin processing procedure affects the adhesive properties of mixed phenol-formaldehyde glues.^54,55^ According to their findings, the properties of kraft lignin-derived phenol-formaldehyde latex outperformed fume-affected lignin-derived phenol-formaldehyde latex.^54^ This kraft lignin prevalence is projected over high-thickness organizations; for example, hydrogen bonding between the parts of kraft lignin and glass transition temperature (*T*_g_) is an essential feature that controls the end product.^54^Fig. 3 depicts the hydrogen bond between lignin's stilbene and amylose.

The broad investigation has proclaimed that *T*_g_ relies upon how much polysaccharides and water are present in lignin and its sub-atomic weight and action.^55,56^ Different synthetic cycles are utilized to deal with lignin from various sources, and each interaction enjoys explicit benefits and damages, and most processes follow an acid or base-synergist device.^56^ In this manner, lignin is subdivided into low sub-atomic weight portions, and its properties are significantly impacted.^56^ In addition, a few researchers have fostered another innovation for handling lignin, the three primary cycles being sulfite, kraft, and modern, alongside different cycles.^55–57^ The sulfite interaction is an acid-catalyzing process generally utilized in pulping, including the α-ether linkage of lignin and the β-ether link.^56,57^ In this technique, the response between free sulfuric acid and lignin produces acidic lignosulfonate, which structures solvent lignosulfonates with cations and separation of lignosulfonates to deliver carbs.^56,57^ Making lignin is a by-product of the art pulping process insoluble in solvents such as water.^57^

In contrast to other lignin, Kraft lignin contains many phenolic groups.^56,57^ The number of phenolic groups increases as the atomic mass of lignin decreases. The ionic strength, temperature, and pH affect the solubility of kraft lignin in water due to bond breaking.^57^ NaOH is widely used to produce wood mash. Soft lignin has several characteristics: a high phenolic and hydroxyl concentration, a relatively low glass transition temperature (*T*_g_), and a low atomic weight. Bio-based lignin in the PLA framework changes the rheological and thermomechanical properties of poly lactic acid (PLA) compounds. Lignin coating on CNCs increases the interfacial contact with the PLA grid, resulting in the upgradation of properties, making them suitable for end-user applications. Amalgams exhibit a considerably larger capacity modulus (*G*′) than clean PLA at all levels of l-CNC in glass and rubber-treated sections in vigorous mechanical tests.^58^ In the presence of l-CNC, a significant improvement in the crystallization behavior of the PLA lattice was also discovered.^58^

Several combinations with different lignin loadings into PLA were achieved using ring-open polymerization of lactide on chosen alkylated lignins. After mixing the copolymer with poly-l-lactide (PLLA), the electron spin interaction is employed to construct a homogeneous nanofiber with a controlled fibre measurement. The PLA–lignin compounds were studied using three distinct cell types: PC12, human mesenchymal undifferentiated organisms (MSCs), and human dermal fibroblasts (HDFs). During mechanical property research, it was discovered that the cleavage and strength of PLA/lignin composites are several times more than that of pure PLA. DPPH testing evaluated the cancer-prevention activity of PLA–lignin copolymers and lignin-based nanofibers. Fine PLLA nanofibers have been found to have limited cancer prevention mobility.^59^ Indeed, even after 72 hours, it achieved only 15.5 ± 6.2% free severe obstruction, much lower than lignin-containing nanofibers. These lignin-based nanofibers are biomaterials to reduce practical difficulties associated with tissue damage or oxidative pressure. PLLA/PLA–lignin biocompatibility has been investigated, and each of the three cell types exhibits reduced metabolic movement on pure PLLA nanofibers due to the polyester-initiated oxidative pressure.^58,59^ All lignin-containing nanofibers had high cell expansion values, suggesting that cancer prevention mobility increases cell feasibility. To reduce cell oxidative pressure locally, such materials can be utilized as tissue-designing platforms, as mentioned.^59^ The cancer prevention action of lignin–PClA copolymers and their electrospun nanofibers were reassessed by DPPH testing. They demonstrated that high lignin stacked copolymer displays high cancer prevention properties. All PLLA/lignin–PCLA nanofibers also exhibit above 70% cancer prevention activity, allowing them to be used in biomaterials and food packaging to solve oxidative pressure^58,59^ concerns.

Even though lignin and its derivative are versatile, the production cost is higher than the conventional method. The isolation of lignin in its original form is difficult; it will always result in by-products.

### 3.4 Starch

Starch is a solid, biodegradable polymer with a low molecular weight^60^ and is the most widely available polymer after cellulose.^3,60^ The fundamental starch sources are rice, wheat, potatoes, and corn. The US is accounted to be a world innovator in starch synthesis. European nations add to worldwide starch creation after the US. These two nations essentially create a large portion of the world's carbs.^60–62^ The regular search is made of a nano-sized particle with a semi-glasslike design. It is associated with the translucent lamellar areas related to one another by amylopectin side chains, bringing about twofold helixes.^61,62^ Such twofold helixes are firmly stuffed, forming the basis of the translucent space. In the amorphous system, the starch particles have a unique chain structure, with a glass-like system so that the starch atoms form a twofold helix state. The amorphous and the crystal structures settle together, changing the arrangement of a ring.^61,62^ Under optical and electron microscopy, the presence of the ring structure which initiates the annular arrangement was seen. Circular granules dominate the morphology of starch. The spherical starch granules' diameter varies from 2 to 100 m, depending on the plant source. Regardless of the source of the starch, the concentration was constant and responded to 1.5 kg m^−1^.^3,61,62^

**Fig. 4:**
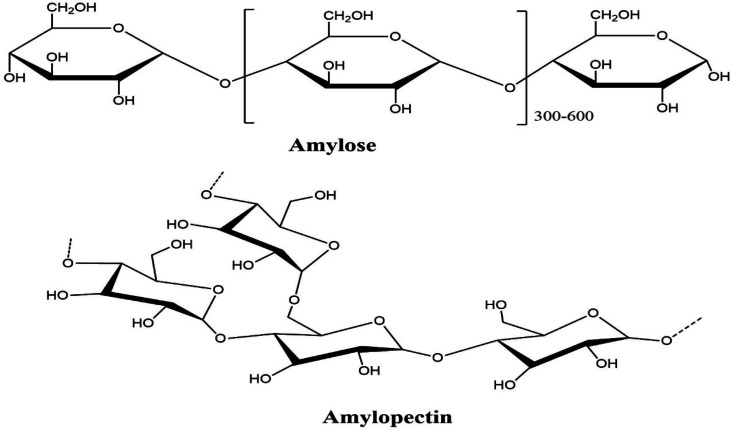
Structure of amylose and amylopectin.

Amylose (Fig. 4), which is mostly straight or slightly extended (1–4) – α-d-glucan has a molecular weight of 105 and 106 g mol^−1^, whereas amylopectin has a molecular weight in the range of 10^7^ to 10^8^ g mol^−1^.^61,62^ Amylopectin is an extended polymer with α-(1–6) linkages that restrict the short length (1–4) – α-d-glucan units. Most starch varieties contain 72–82% amylose, whereas the amylopectin level is between 18 and 28%.^61–63^ Morphologically, the amylopectin part looks glass-like crystalline structure, and the amylose is amorphous or semi-translucent. Consequently, amylose is soluble, while amylopectin is insoluble. In industry, the steps used to remove starch from plant roots include wet crushing, washing, and drying. A white powder-like starch extracted from plant roots is classified as “white flour.” If the white powder is synthetically treated with chemicals, it will have extraordinary properties and is designated “altered starch”.^61,62^

Starch is divided into class A, class B, and class C. The purpose of XRD analysis is to provide a long-term categorization of starch granules based on three separate classifications. Amylopectin chain length has been demonstrated to influence starch biopolymer crystallization.^62^ The authors proposed a model based on a twofold helix insistent on favoring class A and B starches. It was observed that the redesigned feature of the twofold helix structure causes advancement from class A to class B and *vice versa*. It has been observed that each twofold helix structure follows a firm action of A-type, which contains water particles.^62,63^ In class B, however, the pressure is more open to admitting water particles in the significant holes framed by the six double helixes. An XRD diffractogram^63^ indicates that, class C is the combination of class A and B. class C starch is found in bean starch. According to researchers, class B, and class C starch granules are broader than class A.^63^ Distances between classes B and C are between 400 and 500 nm, whereas distances between types A and B are between 25 and 100 nm. Several research articles describing the fundamental features of C-class starch produced from pea seeds are available.^63^ Class C starch contains more polymorphisms than A and B. Starch of grades A and C has been considered more impervious to acid hydrolysis than grades B.^64–66^ The amorphous and shortened branch chains^65^ of amylose and amylopectin account for their translucent organization. In any case, parallel amylopectin chains have not entirely settled, resulting in incomplete starch crystallization.^65^ The proportion of amylose and amylopectin relies upon the plant source utilized for extraction. The balance additionally depends upon the stages engaged with the extraction cycle. Starch displays the most complicated structure that can be known when characterized by a few degrees of association.^63–66^

Starch gelatinization in water at low temperatures can be used as an adhesive or a hardener. Because of its practical problems, using starch on a cutting-edge scale is limited. These typical limitations can be overcome by making suitable structural changes, including physical, substance, and enzymatic strategies.^63–66^ Authentic treatments include heat treatment, reinforcing, pre-gelatinization, high-strain treatment, radiation, and sonication to remove wetness. Engineered methods usually combine with other cross-interfacing reactions, oxidation cycles, and hydrolysis under suitable conditions.^60,62^ A couple of dispersed transmissions have portrayed the improvement of starch-based polymers to decrease natural impacts and increase the extent of uses for biopolymers. The two main qualities which make starch a remarkable biopolymer is (i) the biodegradation of sugars and acids in water and soil and (ii) the environmentally friendly properties.

Moreover, the exceptional properties of starch, like enzymatic changes, lead to the development of new capabilities for starch.^65–68^ Furthermore, crystallization and regressive of starch chains alter thermo-mechanical characteristics.^66^ This kind of limit doesn't satisfy the starch for the packaging application. Though plasticizers can moderate some of the issues, it is impossible to meet all the packaging requirements.^68^

In another report, corn-based starch films were formed with two specific kinds of soothing gums from Zataria multiflora Boise and Menthapulazium plasticizers. Film properties were improved through the emulsification association^68,69^, and an improvement in the water-limiting property for films was found. Despite some changes in physical and mechanical properties, starch-based films are suitable for general packaging applications.^68^ For starch-based films to be applied in the packaging industry,^68^ relative parameters and mechanical properties appeared differently from plastics. As a result, additional mechanical properties are necessary.^68,69^ Making starch-based nanocomposites is the essential strategy for reducing mechanical properties.

Starch is the primary component in starch crystallite, nanocrystal, microcrystalline, and hydrolyzed starch.^68,69^ All of them are glass-like structures and may be produced by hydrolysis. Depending on the crystallinity of sugars,^68,69^ amylopectin chains undergo distortion through the association process. Since starch granules are typically made from hydrocarbons, resulting in a nanocrystal structure.^70^ With starch synthesis in base hydrolysis, the lower side extended areas and the unspecified regions in the starch are separated.^70,71^ Several studies have determined whether sugars from various sources may combine starch nanocrystals and amylose and how starch will influence the final structure. Amylose content in different sources, for instance, corn, wheat, and potato, showed no differentiation in size. Ring sizes of the starch obtained from natural sources with different amylose content are unique in properties.^71^ Starch nanocrystals are expected to be created from starch granules and performed by interrupting the adjacent starch semi-glasslike structure at temperatures below gelatinization.^70,72^ Under these circumstances, the starch is hydrolyzed and degraded into nanoscale glass-like forms.^72^

The hydrolysis temperature is not similar and lies between 35° and 45 °C. This low-temperature range prevents the gelatinization of the starch and the destruction of the starch. Depending on the source and segment pattern of the starch, different sizes and forms of starch nanocrystals can be produced. Various starch sources, such as grain, custard, potato, mung bean, and chicken peas, are employed in destructive hydrolysis to coordinate starch nanocrystals.^70^ The design and morphology of starch nanocrystals depend on source type, crystallization process, nature of amylopectin, and morphology. The morphological characteristics of starch nanocrystals will be affected by the destructive hydrolysis of collaboration of starch granules.^72–74^ Various researchers^72–76^ have demonstrated the platelet-like shape of starch nanocrystals using TEM analysis which has 5–7 nm thick plate-like starch nanocrystals with widths varying from 15 to 40 nm; in some cases, multiple starch sources have resulted in nanocrystals of varying sizes and forms.^72^ Potato starch granules ranging from 40 to 100 nm have been displayed from spherical and grape-like nanocrystals.^75,76^ Much research has been carried out to reduce the size of starch granules from the microscale to the nanoscale using high-pressure homogenization processes.^75,76^

Consequently, scientists observed that starch particle size might be lowered from 3 to 6 m to 10–20 nm.^76,77^ This was only achievable when the starch granules were homogenized at a pressure of 20 ps.^73^ Starch nanocrystals are utilized for the formation of nanocomposites due to their outstanding properties, such as nanoscale platelet-like surface, high crystallinity, low permeability level, and unyielding nature.^75,76^

Experts report a method for getting normal flexible-based nanocomposite upheld with starch nanocrystals from corn. A few reports are available on ordinary versatile supported with starch nanocrystals.^74^ The mechanical and water block characteristics of sorbitol-plasticized Pullan films incorporating waxy maize flour nanocrystals are unsurpassed.^69,70^ Progression is due to the strong bond between starch nanocrystals and polymer lattice. Excellent results for starch nanocrystal-developed/plasticized starch films using glycerol and sorbitol plasticizers have been achieved. Various experts have observed that reducing the starch nanocrystal content of the soy protein network to less than 2% wt boosts the energetic modulus for composite images. In any case, including starch nanocrystals in the soy protein lattice lowered the break-at-break (%) value to ref. 70–72. Experts from several domains demonstrated the extraction and properties of starch nanocrystals from potato sources. SEM and atomic power microscopy are used to explore the formation and limits of nanocomposites (AFM).

The evaluation confirmed that the starch nanocrystals were uniformly disseminated throughout the plastic. When versatility is applied to plastic, homogeneous distribution of starch nanocrystals occurs. The most frequent method for obtaining starch nanocrystals is destructive hydrolysis. Intense movement changes water-insoluble glass-like structures using the destructive hydrolysis system into stable suspensions. The destructive hydrolysis process has been practiced^70,71^ to modify starch and its properties. Several approaches can convert starch cells into nanocrystals, including destructive hydrolysis, enzymatic hydrolysis, and co-crystallization. Starch nanocrystals' main properties, such as biodegradability, remarkable mechanical capabilities, low thickness level, and low susceptibility, make them excellent candidates for coupling with conventional polymer composites. Because starch nanocrystals are polar and hydrophilic, their dispersion level in non-polar fluids is restricted. This results in the mishandling of the starch nanocrystals and the hydrophobic polymers. Fortunately, starch nanocrystals have open surfaces amenable to synthetic derivation and joining processes, altering their surface hydrophobicity and engaging scattering in non-polar fluids.^72^ Several researchers have observed that lowering the surface energy of starch nanocrystals leads them to seek to disperse in polymers. The surface strength of starch nanocrystals enhances their distribution level in the polymer grid.^72,73^ It is now known that the hydroxyl packs in starch nanocrystals may be changed by chemical processes.^72^

The size of the nanoparticles, the shaft form comparable to cellulose, and the chitin and plate-like design for starch nanocrystals are all determined by the polysaccharide source.^74,75^ The formation of glass-like structures from nanofillers derived from these polysaccharides may be an attractive solution for fabricating bio-nanocomposites with high stiffness. Accordingly, the security of packaged things from oxidation, high temperature, and microorganisms can be achieved by using multi-layer structures with different polymers, all of which add to express limits.^74^ The permeability speed of most vapor and gases through a polymer material depends upon its engineered nature and the physico-chemical properties of the vulnerable particles.^76^

Further developing water-holding permeability and oxygen vulnerability in composites/nanocomposites is critical for packaging various food assortments and medication things.^75,76^ Platelet-shaped starch nanocrystals, for example, can shift the scattering approach to more penetrating particles than shaft-shaped cellulose nanocrystals and improve the preventive characteristics of polymer composites.^76,77^ Exacerbation is an essential strategy for managing the existence of unambiguous interchanges between fillers and polymeric structures by increasing crosslinking activity.^75–77^ According to the viewpoint, the interaction of polymeric materials with various solvents is critical because dissolvable grains entering the polymer create changes in the angles and fundamental properties of the material.^75–77^ It is believed that crosslinking modifications prevent starch from expanding. The extent of crosslinking determines the rate of the reaction. Various experts have concentrated on the effects of variables such as polymer framework possibilities (polar or non-polar), the nature of starch nanocrystals, nanocrystal component alterations, various solvents (water, toluene), and reaction times. The results show that water advancement grew rapidly in the pre-submersion period for most pieces containing starch nanocrystals. Ensuing to show up at the most disagreeable level, water confirmation was considered low until a balance was reached. Hence, absorption energy speeds up the first and foremost stages, followed by ingestion level.^73,74^ This connection's diminishing in water maintenance is attributed to the sifting or fragmented appearance of starch nanocrystals into the water, regardless of how starch shows dissolvability.

The connection point between the starch nanocrystals and the polymeric framework, for example, regular elastic, is diminished by expanding the starch area compared with the response time. The primary reason for researching the possibility of polymeric composites is increased biodegradable wrapping materials with improved thermal qualities. Differential filtering calorimetry (DSC) and Dynamic Mechanical Investigation (DMI) do not entirely demonstrate warm behavior, including the assurance of glass progress temperature (*T*_g_) (DMA). *T*_g_ is generally the temperature-dependent refine point of the bend region of the last digression in the DMA method, which focuses on polymer chain movement through-unwinding at the atomic level (tan). The thermal stability of these polymer cells can be analyzed by utilizing thermogravimetric investigation (TGA).^76^

It has been noted that nanocomposites made up of nanoparticles/nanofillers mostly show better thermal properties.^77,78^ Interestingly, the properties of polysaccharide nanocrystals rely upon the source, the combined cycle, and the kind of surface change.^77^ For instance, sulfuric acid hydrolysis prompts the surface covering of nanocrystals with sulfate ester linkage of acidic nature and decreases the temperature step by step. Lately, nanocrystals from starch have displayed predominant thermal properties and rare temperatures.^77,78^ The information regarding the crystallization of starch granules is significant for the massive scope for forming starch nanocrystals.^77,78^ The polymorphism of α-glucans is the primary component of the transparent system in starch granules. Because regular starch granules contain glass-like zones, XRD can detect their existence. The presence of the X-beam diffractogram relies upon how much water is present in the starch granules, and the higher the starch hydration content, the lesser the diffraction design.^76–78^ Like this, hydration is a significant and fundamental variable for linking starch-like locales. The progress from glass-like to open-ended structure is mainly brought about by gelatinization in the water between 60 and 70 °C.

Starch has a translucent substance of 15–45%, and XRD tests A, B, and C^77^ affirm the various sorts of carbohydrates. Because of its extraordinary nature and minimal expense, it is viewed as a suitable polymer for assembling biodegradable nanoparticles.

Polyhedral granules are nanocrystals derived from maize flour, although their form varies depending on the sugar.^76–78^ The biggest nanocrystals with an average granular size of 41.3 m were removed from potato flour containing vegetable starch. Curiously, all starch nanocrystals delivered by acid hydrolysis show a round shape no matter their starting point.^76–78^ The salient properties of starch, such as renewability, binding ability, duration, and film-forming ability, have increased their use in the therapeutic field in the form of disintegrants and lubricants. Poor mechanical strength and high moisture content are the main demerits of starch-based materials.

## 4 Metal nanocomposites

In the modern era, metal nanocomposites have proven their efficiency in ecological applications. Environmental change, water contamination, and unsafe gases are increasing significantly due to the prolonged use of petroleum products and plastic items. In general, unsustainability leads to air, water, and soil pollution. The utilization of metal nanoparticles, nanocomposites, and metal–organic frameworks to identify contaminants was progressed to eliminate the pollution.

Image-initiated charge barrier should produce a more prominent relationship between titanium nanotubes (dynamite) and graphene, improving photocatalytic activity.^78^ The limitations associated with pure TiO_2_ nanomaterials will be overcome by combining TiO_2_ with metal oxides or sulfides, namely ZnO/TiO_2_, CuO/TiO_2_, SnO_2_/TiO_2_, Discs/TiO_2_, and ZnS/TiO_2_ – in UV and visible light.^78,79^ Experts were familiarized with hydrogenation, metal, and non-metal doping and knew about the small semiconductor hole and advanced productivity in daylight utilization.^80^ These methodologies have shown progress in photocatalytic activity.^79^ Three kinds of dynamic nanomaterials have been created utilizing crude compounds. They can be effectively created using iron oxides or iron sulfides (ZnO) to get ZnO-dynamite, and Cds TiO_2_ strengthens that work.^81,82^ Graphene oxide (GO) and graphene (GR) are carbonaceous materials that are ideally appropriate to act as stimuli.^79^ Graphene oxide can increase the dispersion of the TiO_2_ impulse and decrease the tendency to re-gather an electron–preamble pair. Both graphene oxide and graphene may be combined with TiO_2_ to form graphene-TiO_2_/GR or graphene oxide-TiO_2_/GO composites that can isolate water and reduce wastewater pollution.^79^

TiO_2_ nanotubes with upgraded reactant movement can be effortlessly created under exact circumstances; a few examinations are in progress to improve additional properties.^79,82–84^ Titanium nanotube (dynamite) links with TiO_2_ nanoparticles, with enhanced properties like high conductivity, huge region, good mechanical properties and gases, and optimal optical properties.^79^ To improve dynamite action, it is proposed to build the adsorption of reactive and light atoms.^85,86^ Furthermore, an enormous proportion of dynamite nanostructure and GR ought to further develop the charge partition of picture.^86^ All of the above factors will increase the photocatalytic action of TiO_2_ nanoparticles.^79^

## 5 Nanoencapsulation and their green properties

**Fig. 5:**
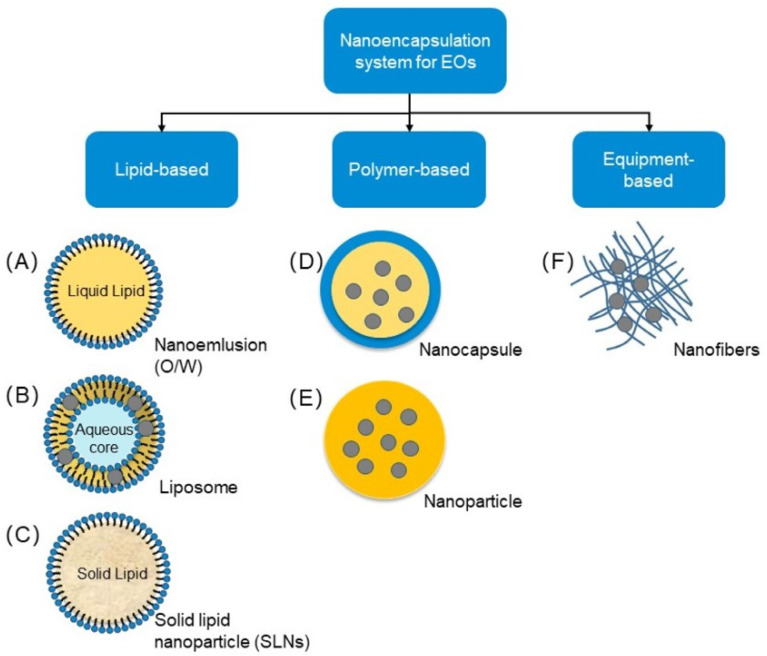
Types of nanoencapsulation (Reproduced from ref. 88 with permission from MDPI, copyright 2021).

Many nanoparticle-based frameworks are created from explicit parts adequate to combine micronutrients.^82^ Novel nanoencapsulation approaches are performed to give the well-being of bioactive mixtures, for example, nutrients, cell reinforcements, lipids, and proteins.^82^ The system is chiefly utilized to create food with dynamic properties.^82,85^ These innovations are promising advancements regarding nutrition and general well-being.^82,85^ A few nanocarriers combined with nutraceuticals for dietary frameworks have been created.^85,87^ The different encapsulation methods are shown in Fig. 5.

Bio-based stage change materials (bio-PCM) have recently been successfully extricated into ultra-fine filaments using coaxial electrospinning.^82^ Regular soy wax has been used as a bio-PCM for heat capacity and a covering shell component in polyurethane (PU) epitome.^82,85^ Bio-PCM strands have been studied using various microscopy and spectroscopic techniques. Data shows that coaxial electrospinning prompts a homogeneous wax conveyance at the center of the fiber and a reliable fiber morphology with a shell design.^85^ Fibrous structures show heat stock up and deliver thermo-control properties.^85^ Thermal properties don't change after many heating–cooling cycles, demonstrating that the composite fiber has remarkable heat strength and security.^82,85^ Microencapsulation of plant-derived bio-based polylactic acid (PLA) in oil and water emulsification was also investigated. Fourier changes, infrared spectroscopy, and scanning electron microscopy confirmed the presence of acid in PLA shells.^86^ The thermal characteristics, thermal stability, and change in material microcapsules have been studied using differential calorimetry.^86^ Through a few parametric tests, the stage change relies upon the impact of the material as well as the size, oil stage, water stage proportion, and surfactant refinements on the morphology, molecular features, and thermal properties.^86,87^ Precise trial results have shown that PVA is an excellent emulsifier.^89^

Moreover, there is a suitable PVA material to diminish the particular sizes of the microphase in the material.^89^ Considering the amount of PVA, the emulsifier particles will interface with one another to surround mycelium.^80,90^ This outcome incorporates tiny microspheres on the upper surface of the microphase material with the more extensive.^80,86^ The SEM microstructure displays minute phase change materials containing 0.4, 0.6, and 0.8 g of palmitic acid. It has been observed that small phase change is unaffected by material size and surface, assuming the wax content is high.^86^ Furthermore, as the small phase changes, materials display their round shape, and some irregular surface appearance has been seen with more modest infinitesimal circles.^82–90^

The electrohydrodynamic fabricating technique (EHDP) is utilized to create ordinary aloe (AV, aloe barbadensis mill operator) using engineered polymers, for example, polyvinylpyrrolidone (PVP) as well as poly(vinyl liquor) (PVOH) together. Typically delivered polymers incorporate grain starch (BS), whey protein concentrate (WPC), and maltodextrin.^87,91^ Avi leaf juice has been utilized as a water-based substance for EHDP, so the properties of biopolymer solvents are investigated, as their impact on interaction.^91^ The morphological assessment acted in the past segments relies upon the accumulating conditions and the idea of the engineered polymers (principally made of fiber-like courses of action).^91^ Regular size ranges from 100 nm to three m. Because of their particular and ideal morphology, high fiber size in nanofiber shape, PVP, and WPC of the nanocapsule are investigated AV strength against bright (UV) light conditions. Infrared (FTIR) spectroscopy exposed the fascinating epitome of AVs in biopolymer networks, which show fantastic synthetic networks with bioactive substances.^90,92^

## 6 Nature-induced hydrogel

**Fig. 6:**
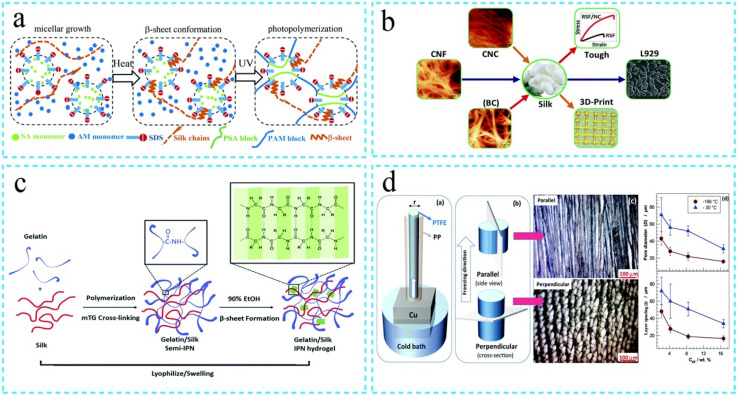
Steps involved in the synthesis of hydrogel (a) double network hydrogel (b) mechanical properties of the hydrogels (c) crosslinked structure of hydrogels (d) crosslinking process (Reproduced from ref. 93 with permission from Royal Society of Chemistry, copyright 2021).

Hydrogels are polymeric materials with hydrophilic qualities that are useful in various industries.^80,89^ For over fifty years, hydrogels have been used because of their attractive physicochemical properties^89^ due to the three-layered network, which shapes the hydrogel structure.^89^ Hydrogels' dominant feature is the capacity to adapt and hold enormous proportions of watery fluid.^80^ It results in a hydrophilic cross-associated network that can keep a three-layered structure even in the extended state without dissolving.^80^ The hydrogel structures (Fig. 6) have permanent covalent links between the polymer chains of the hydrogel.^80,89^ Of course, in real, crosslinked hydrogels, known as natural hydrogels, the polymer chains combine through electrostatic hydrogen bonding and van der Waals force.^80,89^ Due to their reversible nature, real hydrogels separate by losing the open links that lead to crosslinking.^80,89^ Similar to pH, ionic strength, and electric field, variations in the external medium separate real hydrogels.^80^

Notwithstanding the way that there are contrasts among compound and genuine hydrogel advancement pathways, it is doable to get hydrogels in many designs, for instance, circles, chambers, motion pictures, and layers.^80,89^ Moreover, hydrogels can similarly be made in huge scope, short and nanoscale assessments. Hydrogels have been solely employed for liquid ingestion/support for the previous two decades. The capability of these potential class materials is now being used in a broad range of current, creative, and biotechnology fields. The current condition is achieved *via* the strategy of another class of intelligent hydrogels, which may supply various reactions (such as volume, porosity, and mechanical alterations) to external and internal assistance.^80,89^ Despite these advances, various polymers have radically altered their properties and use.^80^ Biopolymers (such as polysaccharides and proteins) have fascinating and attractive physicochemical properties (such as biocompatibility, biodegradability, and non-destructiveness) that enhance their use in different fields.^10,89^ For example, hydrogels loaded with biopolymers (essential polysaccharides) have found great applicability as biometrics. In addition, the attractive properties of polysaccharides due to the vast combination of supportive moieties (–COOH, NH_2_, NHOCCH_3_, and OSO_3_H) can be crosslinked by responding with the coupling moiety. Of course, it allows the polysaccharide to connect crosslinking or polymer chains into the spine.^90,91^ Macromolecules can create polysaccharide-based hydrogels with polyelectrolyte structures through reverse electrical charges.^91^ As inspected previously, starch shows all supportive properties expected to prepare natural hydrogels.^89–91^ Collecting starch-based hydrogels using various procedures has been portrayed in reports and review articles.^90,91^ In general, the critical technique for manufacturing starch-based hydrogels is based on the reaction of the hydroxyl mobility of the starch with the bi-or different combinations.

Recently, glutaraldehyde and epichlorohydrin have been the most often utilized chemicals.^80^ Regardless of this approach, employing a coupling agent is not required when manufacturing hydrogels for biomedical reasons.^87,91^ The coupling agent could show some degree of toxicity, lessening the suitable extent of the compound hydrogels.^87^ Coordinating excessive reactions of unsaturated monomers bearing carbon–carbon two-fold structure with starch or starch-based macromonomers is a robust approach usually used to predict the inconvenient issue of crosslinkers. This approach is frequently used to transport superabsorbent hydrogels.^89,90^ Copolymerization and crosslinking of starch with vinyl-functionalized monomers have been described.^89–91^ Most hydrogels synthesized using this method show semi-network properties.^80,90^ IUPAC describes fragmentary IPN as a polymeric substance containing one straight or extended polymer at the atomic scale. The semi-interpenetrate network has interpenetrating bonding. It is due to the direct or extended polymers prepared from the semi-interpenetrating bonding can be disengaged from the polymer networks.

The primary benefits of hydrogels are (I) fluid intake limitation (development), (II) network-like morphology, (III) sub-nuclear development, and (IV) mechanical qualities. These components are quantitatively orchestrated using an infinite number of polymers.^87^ Most starch-based hydrogel structures, as previously stated, are related to two fundamental elements: (I) the polymer mixture and (ii) the process used to approach the hydrogel.^80,89,90^ These variables influence the quantity, porosity, and location of hydrophilic groups within or on the surface of the hydrogel group. For example, the hydrophilicity of the starch-based hydrogel lattice determines the fluid retention limit. Several restrictions govern variations in temperature or pH, such as salinity and the medium's ionic strength. Starch-based hydrogels, often made with hydrophilic polymers/monomers, may consume and retain fluids, classifying them as super-retentive.^80^ In this manner, it requires the expansion of starch to more hydrophilic polymers to deliver materials with higher fluid limits.^91^ In polymer research, hydrogels have evolved into materials with outstanding properties and numerous prospective applications, ranging from soil preservation and tissue design, drug transmission frameworks, and reproduced polymers.^80,87,89–91^ As a result, hydrogels and hydrogel compounds continue to be attractive topics in the physical study.^80,87,89–91^ Hydrogel composites are considered micro and nano-sized particles with advanced thermal, mechanical, and optical properties, water intake limit, and solubility discharge rate in their production. Inorganic salts, hydroxyapatite, metallic and attractive nanoparticles, carbon nanotubes, polysaccharide nanocrystals, and quantum dots are support materials.^91^ Soil modification should be possible by displacing the metallic cations in the old soils with natural cations, creating altered surfaces between the polymers and the dirt.^80,87,89,90^ Nonetheless, a few nanogels have been accounted for; the region is in the beginning phases of improvement. Hydrogel-based micronutrients produced using polysaccharides/starch are mainly utilized in clinical applications.^92^

## 7 The versatility of green nanocomposites

Unlike different micronutrients, it is feasible to incorporate green micronutrients or bionanomaterials from plant and natural sources.^90,91^ The most well-known strategies used to make nanocrystals are mechanical functionalization and acid hydrolysis.^90^ Acid hydrolysis assists with eliminating/disintegrating lower demand areas so the profoundly insoluble high translucent structure can be changed over into a steady suspension by mechanical force.^90^ For years, various research has been conducted to design bio-composites by joining/reinforcing bio-nanomaterials in diverse polymer lattices.^90,91^ New improvements in the manufacture and arrangement of various polymer nanocomposites are being discussed in the current domain.^90^

**Fig. 7:**
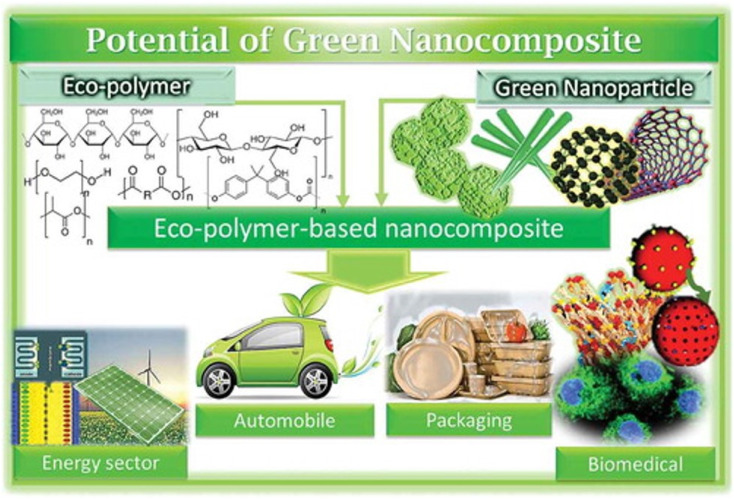
Applications of polymer-based nanocomposites (Reproduced from ref. 94 with permission from Taylor & Francis, copyright 2021).

Biopolymer nanocomposites have emerged as an advanced topic of investigation in nanotechnology, attracting considerable attention in various fields (Fig. 7) during the last ten years. Difficulties in this investigation include (i) successful detachment pathways for separating nano-fortifications from sustainable assets, (ii) accomplishing similarity between nano-fortifications and polymer grid, and (iii) proper analysis. Furthermore, energy consumption and cost are crucial variables in commercializing bio-nanocomposite-based substances. It is worth noting that adding nano-support to the polymer network is the most widely used technique for working on the characteristics of biopolymers.^91^ This strategy has been set up for quite a long time, yet it is still in the improvement stage.

Chitin shows a high proportion when its fibres are utilized to build fillers for developing polycaprolactone (PCL) based nanocomposites. Chitin fibres from riftia tubes have been accounted for equal ends.^92^ To design films with an amorphous polymer framework, manufacturing processes like hot squeezing and freeze-drying should be used (styrene-co-butyl acrylate). Polyvinyl alcohol (PVA) films with chitin whiskers have thermal characteristics.

A 2.96 wt% chitin hair focus produces the most excellent stiffness. As the convergence of chitin hair expanded further, the augmentation at-break (%) diminished.^92,95,96^ An X-beam diffractogram and FTIR spectroscopy show the presence of chitin in the PVA grid.^97^ The existence of chitin in the PVA lattice was confirmed by the occurrence of a strong peak at 1144 cm^−1^. The properties of chitin-filled regular elastic composites have been assessed.^95^ The presence of an inflexible association of chitin hairs in the regular flexible lattice was affirmed.^97^ The existence of chitin's inflexible 3D system in the stable elastic network is confirmed by various factors like elasticity, diffusion coefficient, and weight reduction.^98^

Starch nanocrystal's fundamental and morphological properties developed regular elastic combinations.^97,98^ Freeze-dried starch nanocrystals showed typical diffraction designs. There is proof of an expansion in the crystallization of specific flexible structures compared with starch nanocrystals.^97^ Oxygen and water emission penetrability rates for regular elastic/starch nanocrystals are additionally being examined. Blended films have a border line impact on both oxygen and water emission. Reduced cost and expanded water accommodation properties of regular elastic/starch nanocrystals have been found. The saturation power is 10 wt%,^99,100^ for developing hydrogen bonding networks between starch nanocrystals in the regular elastic grid. Several studies on the characteristics of PLA nanocomposites have been published.

Nonetheless, evaluation of the antibacterial characteristics of PLA nanocomposite films is limited.^97,99,100^ The effect of nano clays on the antibacterial properties of PLA films made using the well-known technique has been investigated. For the KENAF fiber-based PLA composite, it was seen that the controllable and flexible strength in the KENAF fiber content expanded by up to 50 wt%. PLA composite films are exposed to a biodegradability assessment for over 25 days. Toward the finish of the biodegradation study,^100^ there was proof that PLA compounds showed a 38% decrease in weight. The impact of fiber content on the properties of PLA intensifies has been explored. Upgrades in mechanical properties, such as^57,58^ rigidity and twisting strength, were found for PLA amalgams when the fibre content was kept at 45–65 wt%. The impact of Kenaf fiber content on the mechanical properties of PLA composites^57,58,100^ has been anticipated. The PLA network^57,58^ and 30 wt% Kenaf fiber soluble composite altered mechanical properties.^98^ An improvement in mechanical properties was found at 25 wt% in the polylactic acid (PLA) lattice. Because of flax-built-up PLA amalgams, the effect of strength was viewed as lower than that of clean PLA.^57,58^ Infusion from PLA composites with 25 wt% SEB (steam-exploded bamboo) strands shows predominant mechanical properties. The strength and solidness of PLA/SEB strands were double that of PLA.^57,58^

## 8 Conclusion

The abundance, replicability, and desirable modifications in the green nanobiopolymers open a new window in the branch of interdisciplinary sciences, where its applications and utility can be exploited as per the need of a particular problem or situation. Green nanobiopolymers can be the future in various aspects of textile, polymers, medical, and healthcare sectors. Polylactic acids, lignin, chitin, polylactones, cellulose, nanocellulose, *etc.*, are suitable green nanobiopolymers. The process of nanoencapsulation also offers many nutritious advantages through the fortification and proper release of bioactive compounds. Here in this review, we have outlined different green nanobiopolymers and the processes involved in their preparations.

Nanocellulose is a potential green nanobiomaterial with tailored-made properties that find various applications in the industrial and medical fields. With the development of low-cost nanocellulose, many new applications will be explored. Synthesis and fabrication of eco-friendly polymeric nanoparticles are emerging research arena and finds applications in numerous field. A feasible and effective synchronization of greener polymeric nanocomposite in targeted drug delivery systems will minimize the adverse effect on human health. Consequently, budding researchers should find a solution to fulfill the gap between laboratory level to large-scale production and introduce the effectual product into the market.

Green nanobiopolymer products will be biodegradable and perishable; thus, it will reduce the toxic effects of many synthetic materials (*viz.*, plastics) that are non-degradable and release harmful by-products into the environment during their manufacturing or disposal processes. Green nanobiopolymer, their preparation processes, and the present applications, which are expected to become an essential part of the human lifestyle-with an environmentally friendly approach, are discussed in the present article. Hence, this review will help to understand and identify the importance of green nanobiopolymers as a futuristic material with many applications.

## Authors contribution

P. C. and K. R. B. S.: conceptualization, data curation, investigation, resources, validation, visualization, writing – original draft. A. N. and R. G. K.: data curation, validation, visualization, writing – original draft. J. S., J. M., and R. P. S.: conceptualization, validation, project administration, supervision, writing – review & editing.

## Conflicts of interest

The authors declare no conflict of interest for this work.

## Supplementary Material

## References

[cit1] Mishra R. K., Sabu A., Tiwari S. K. (2018). Materials chemistry and the futurist eco-friendly applications of nanocellulose: status and prospect. J. Saudi Chem. Soc..

[cit2] Zinoviadou K. G. , Kastanas P., Gougouli M. and Biliaderis C. G., Innovative bio-based materials for packaging sustainability, Innov. Strateg. Food Ind. Tools Implementation, 2nd end, 2022, pp. 173–192

[cit3] Reddy M. M., Vivekanandhan S., Misra M., Bhatia S. K., Mohanty A. K. (2013). Biobased plastics and bionanocomposites: current status and future opportunities. Prog. Polym. Sci..

[cit4] Attaran S. A., Hassan A., Wahit M. U. (2015). Materials for food packaging applications based on bio-based polymer nanocomposites: a review. J. Thermoplast. Compos. Mater..

[cit5] Mohiuddin S. H. M., Kumar B., Mohiuddin S. H. M. (2017). Biopolymer Composites in Photovoltaics and Photodetectors. Biopolym. Compos. Electron..

[cit6] Tang X. Z., Kumar P., Alavi S., Sandeep K. P. (2012). Recent Advances in Biopolymers and Biopolymer-Based Nanocomposites for Food Packaging Materials. Crit. Rev. Food Sci. Nutr..

[cit7] Mekonnen T., Mussone P., Khalil H., Bressler D. (2013). Progress in bio-based plastics and plasticizing modifications. J. Mater. Chem. A.

[cit8] Kabasci S. , Bio-Based Plastics – Introduction, Bio-Based Plastics: Materials and Applications, 1st edn, 2014, pp. 1–7

[cit9] Mokhtarzadeh A., Alibakhshi A., Hejazi M., Omidi Y., Ezzati Nazhad Dolatabadi J. (2016). Bacterial-derived biopolymers: Advanced natural nanomaterials for drug delivery and tissue engineering. TrAC, Trends Anal. Chem..

[cit10] Ulery B. D., Nair L. S., Laurencin C. T. (2011). Biomedical applications of biodegradable polymers. J. Polym. Sci., Part B: Polym. Phys..

[cit11] Harane R. S., Mehra N. R., Tayade P. B., Adivarekar R. V. (2014). A facile energy and water-conserving process for cotton dyeing. Int. J. Energy Environ. Eng..

[cit12] Irmak S., Kurtuluş M., Hasanoĝlu Hesenov A., Erbatur O. (2013). Gasification efficiencies of cellulose, hemicellulose and lignin fractions of biomass in aqueous media by using Pt on activated carbon catalyst. Biomass Bioenergy.

[cit13] Kalia S., Boufi S., Celli A., Kango S. (2013). Nanofibrillated cellulose: surface modification and potential applications. Colloid Polym. Sci..

[cit14] Mokhothu T. H., John M. J. (2015). Review on hygroscopic aging of cellulose fibres and their biocomposites. Carbohydr. Polym..

[cit15] Ding S. Y., Zhao S., Zeng Y. (2013). Size, shape, and arrangement of native cellulose fibrils in maize cell walls. Cellulose.

[cit16] French A. D. (2013). Idealized powder diffraction patterns for cellulose polymorphs. Cellulose.

[cit17] Lee C. M., Mittal A., Barnette A. L., Kafle K., Park Y. B., Shin H., Johnson D. K., Park S., Kim S. H. (2013). Cellulose polymorphism study with sum-frequency-generation (SFG) vibration spectroscopy: identification of exocyclic CH_2_OH conformation and chain orientation. Cellulose.

[cit18] Barnette A. L., Lee C., Bradley L. C., Schreiner E. P., Park Y. B., Shin H., Cosgrove D. J., Park S., Kim S. H. (2012). Quantification of crystalline cellulose in lignocellulosic biomass using sum frequency generation (SFG) vibration spectroscopy and comparison with other analytical methods. Carbohydr. Polym..

[cit19] Ventura C., Pinto F., Lourenço A. F., Ferreira P. J.
T., Louro H., Silva M. J. (2020). On the toxicity of cellulose nanocrystals and nanofibrils in animal and cellular models. Cellulose.

[cit20] Barnette A. L., Bradley L. C., Veres B. D., Schreiner E. P., Park Y. B., Park J., Park S., Kim S. H. (2011). Selective detection of crystalline cellulose in plant cell walls with sum-frequency-generation (SFG) vibration spectroscopy. Biomacromolecules.

[cit21] Moran-Mirabal J. M. (2013). The study of cell wall structure and cellulose–cellulase interactions through fluorescence microscopy. Cellulose.

[cit22] Moran-Mirabal J. M. (2013). Advanced-Microscopy Techniques for the Characterization of Cellulose Structure and Cellulose-Cellulase Interactions. Cellul. – Fundam. Asp..

[cit23] Kim J. H., Shim B. S., Kim H. S., Lee Y. J., Min S. K., Jang D., Abas Z., Kim J. (2015). Review of nanocellulose for sustainable future materials. Int. J. Precis. Eng. Manuf..

[cit24] Nechyporchuk O., Belgacem M. N., Bras J. (2016). Production of cellulose nanofibrils: a review of recent advances. Ind. Crops Prod..

[cit25] Zhang T., Zheng Y., Cosgrove D. J. (2016). Spatial organization of cellulose microfibrils and matrix polysaccharides in primary plant cell walls as imaged by multichannel atomic force microscopy. Plant J..

[cit26] Wang M., Miao X., Li H., Chen C. (2022). Effect of length of cellulose nanofibers on mechanical reinforcement of polyvinyl alcohol. Polymers.

[cit27] Habibi Y., Goffin A. L., Schiltz N., Duquesne E., Dubois P., Dufresne A. (2008). Bionanocomposites based on poly(ε-caprolactone)-grafted cellulose nanocrystals by ring-opening polymerization. J. Mater. Chem..

[cit28] Ioelovich M. (2012). Optimal Conditions for Isolation of Nanocrystalline Cellulose Particles. Nanosci. Nanotechnol..

[cit29] Jahanbaani A. R., Behzad T., Borhani S., Darvanjooghi M. H. K. (2016). Electrospinning of cellulose nanofibers mat for laminated epoxy composite production. Fibers Polym..

[cit30] Buchholt M. C. H. C., Christensen T. M. I. E., Fallesen B., Ralet J. F. T. (2004). Preparation and properties of enzymatically and chemically modified sugar beet pectins. Carbohydr. Polym..

[cit31] Rojas J., Bedoya M., Ciro Y. (2015). Current Trends in the Production of Cellulose Nanoparticles and Nanocomposites for Biomedical Applications. Cellul. Fundam. Asp. Curr. Trends.

[cit32] Carrillo C. A., Laine J., Rojas O. J. (2014). Microemulsion systems for fiber deconstruction into cellulose nanofibrils. ACS Appl. Mater. Interfaces.

[cit33] Sipponen M. H., Lange H., Crestini C., Henn A., Österberg M. (2019). Lignin for Nano- and Microscaled Carrier Systems: Applications, Trends, and Challenges. ChemSusChem.

[cit34] Zhang Y., Nypelö T., Salas C., Arboleda J., Hoeger I. C., Rojas O. J. (2013). Cellulose nanofibrils: from strong materials to bioactive surfaces. J. Renewable Mater..

[cit35] Kluge J., Muhrer G., Mazzotti M. (2012). High pressure homogenization of pharmaceutical solids. J. Supercrit. Fluids.

[cit36] Van De Voorde M. , Industrialization – Large-Scale Production of Nanomaterials/Components, Nano-Micro Interface Bridg. Micro Nano Worlds, 2nd edn, 2015, vol. 2, pp. 677–684

[cit37] Peng Y., Gardner D. J., Han Y. (2011). Drying cellulose nanofibrils: in search of a suitable method. Cellulose.

[cit38] Jarrah N. A., Li F., Van Ommen J. G., Lefferts L. (2005). Immobilization of a layer of carbon nanofibres (CNFs) on Ni foam: a new structured catalyst support. J. Mater. Chem..

[cit39] Hu C., Zhao Y., Li K., Zhu J. Y., Gleisner R. (2015). Optimizing cellulose fibrillation for the production of cellulose nanofibrils by a disk grinder. Holzforschung.

[cit40] Josset S., Orsolini P., Siqueira G., Tejado A., Tingaut P., Zimmermann T. (2014). Energy consumption of the nanofibrillation of bleached pulp, wheat straw and recycled newspaper through a grinding process. Nord. Pulp Pap. Res. J..

[cit41] Government N. B. I. R. M., Okeke E. T., Odera R. S., Ani A. K., Thaddaeus J. (2020). Significance of alkaline treatment on the composition of mango seed shell fiber for polymer composite application. Indian J. Sci. Technol..

[cit42] Alemdar A., Sain M. (2008). Isolation and characterization of nanofibers from agricultural residues – Wheat straw and soy hulls. Bioresour. Technol..

[cit43] Villares A., Moreau C., Bennati Granier C., Garajova S., Foucat L., Falourd X., Saake B., Berrin J. G., Cathala B. (2017). Lytic polysaccharide monooxygenases disrupt the cellulose fibers structure. Sci. Rep..

[cit44] Redlinger-Pohn J. D., Grabner M., Zauner P., Radl S. (2016). Separation of cellulose fibres from pulp suspension by froth flotation fractionation. Sep. Purif. Technol..

[cit45] Vasconcelos N. F., Feitosa J. P. A., da Gama F. M. P., Morais J. P. S., Andrade F. K., de Souza Filho M. d. S. M., Rosa M. d. F. (2017). Bacterial cellulose nanocrystals produced under different hydrolysis conditions: Properties and morphological features. Carbohydr. Polym..

[cit46] Yeul V. S., Rayalu S. S. (2012). Unprecedented Chitin and Chitosan: A Chemical Overview. J. Polym. Environ..

[cit47] Zuber M., Zia K. M., Barikani M. (2013). Chitin and chitosan based blends, composites and nanocomposites. Adv. Nat..

[cit48] Boujemaoui A., Mongkhontreerat S., Malmström E., Carlmark A. (2015). Preparation and characterization of functionalized cellulose nanocrystals. Carbohydr. Polym..

[cit49] Novo L. P., Bras J., García A., Belgacem N., Curvelo A. A. S. (2015). Subcritical Water: A Method for Green Production of Cellulose Nanocrystals. ACS Sustainable Chem. Eng..

[cit50] Poerio A., Petit C., Jehl J. P., Arab-Tehrany E., Mano J. F., Cleymand F. (2020). Extraction and physicochemical characterization of chitin from cicada orni sloughs of the south-eastern French mediterranean basin. Molecules.

[cit51] Rinaudo M. (2006). Chitin and chitosan: Properties and applications. Prog. Polym. Sci..

[cit52] Rinaudo M. (2006). Chitin and chitosan: Properties and applications. Prog. Polym. Sci..

[cit53] Paul T., Halder S. K., Das A., Ghosh K., Mandal A., Payra P., Barman P., Das Mohapatra P. K., Pati B. R., Mondal K. C. (2015). Production of chitin and bioactive materials from Black tiger shrimp (Penaeus monodon) shell waste by the treatment of bacterial protease cocktail. 3 Biotech.

[cit54] Ghaffar S. H., Fan M. (2014). Lignin in straw and its applications as an adhesive. Int. J. Adhes. Adhes..

[cit55] Sain M., Panthapulakkal S. (2006). Bioprocess preparation of wheat straw fibers and their characterization. Ind. Crops Prod..

[cit56] Leskinen T., Kelley S. S., Argyropoulos D. S. (2015). Refining of Ethanol Biorefinery Residues to Isolate Value Added Lignins. ACS Sustainable Chem. Eng..

[cit57] Leskinen T., Kelley S. S., Argyropoulos D. S. (2015). Refining of Ethanol Biorefinery Residues to Isolate Value Added Lignins. ACS Sustainable Chem. Eng..

[cit58] Sukmawan R., Takagi H., Nakagaito A. N. (2016). Strength evaluation of cross-ply green composite laminates reinforced by bamboo fiber. Composites, Part B.

[cit59] Kai D., Ren W., Tian L., Chee P. L., Liu Y., Ramakrishna S., Loh X. J. (2016). Engineering Poly(lactide)-Lignin Nanofibers with Antioxidant Activity for Biomedical Application. ACS Sustainable Chem. Eng..

[cit60] Wang S., Li C., Copeland L., Niu Q., Wang S. (2015). Starch Retrogradation: A Comprehensive Review. Compr. Rev. Food Sci. Food Saf..

[cit61] Sonnewald U., Kossmann J. (2013). Starches—from current models to genetic engineering. Plant Biotechnol. J..

[cit62] Sonnewald U., Kossmann J. (2013). Starches—from current models to genetic engineering. Plant Biotechnol. J..

[cit63] Grommers H. E. and van der Krogt D. A., Potato Starch: Production, Modifications and Uses, Starch, 3rd edn, 2009, pp. 511–539

[cit64] Lin L., Guo D., Zhao L., Zhang X., Wang J., Zhang F., Wei C. (2016). Comparative structure of starches from high-amylose maize inbred lines and their hybrids. Food Hydrocolloids.

[cit65] Lin L., Guo D., Zhao L., Zhang X., Wang J., Zhang F., Wei C. (2016). Comparative structure of starches from high-amylose maize inbred lines and their hybrids. Food Hydrocolloids.

[cit66] Mahmood K., Kamilah H., Shang P. L., Sulaiman S., Ariffin F., Alias A. K. (2017). A review: Interaction of starch/non-starch hydrocolloid blending and the recent food applications. Food Biosci..

[cit67] Miranda C. S., Ferreira M. S., Magalhães M. T., Santos W. J., Oliveira J. C., Silva J. B. A., José N. M. (2015). Mechanical, Thermal and Barrier Properties of Starch-based Films Plasticized with Glycerol and Lignin and Reinforced with Cellulose Nanocrystals. Mater. Today: Proc..

[cit68] Miranda C. S., Ferreira M. S., Magalhães M. T., Bispo A. P. G., Oliveira J. C., Silva J. B. A., José N. M. (2015). Starch-based Films Plasticized with Glycerol and Lignin from Piassava Fiber Reinforced with Nanocrystals from Eucalyptus. Mater. Today: Proc..

[cit69] Miranda C. S., Ferreira M. S., Magalhães M. T., Bispo A. P. G., Oliveira J. C., Silva J. B. A., José N. M. (2015). Starch-based Films Plasticized with Glycerol and Lignin from Piassava Fiber Reinforced with Nanocrystals from Eucalyptus. Mater. Today: Proc..

[cit70] Lecorre D., Vahanian E., Dufresne A., Bras J. (2012). Enzymatic pre-treatment for preparing starch nanocrystals. Biomacromolecules.

[cit71] Hao Y., Chen Y., Li Q., Gao Q. (2018). Preparation of starch nanocrystals through enzymatic pre-treatment from waxy potato starch. Carbohydr. Polym..

[cit72] Zhou J., Tong J., Su X., Ren L. (2016). Hydrophobic starch nanocrystals preparations through crosslinking modification using citric acid. Int. J. Biol. Macromol..

[cit73] Li Z., Liu W., Gu Z., Li C., Hong Y., Cheng L. (2015). The effect of starch concentration on the gelatinization and liquefaction of corn starch. Food Hydrocolloids.

[cit74] Li Z., Liu W., Gu Z., Li C., Hong Y., Cheng L. (2015). The effect of starch concentration on the gelatinization and liquefaction of corn starch. Food Hydrocolloids.

[cit75] Haaj S. B., Magnin A., Pétrier C., Boufi S. (2013). Starch nanoparticles formation via high power ultrasonication. Carbohydr. Polym..

[cit76] Chen P., Xie F., Zhao L., Qiao Q., Liu X. (2017). Effect of acid hydrolysis on the multi-scale structure change of starch with different amylose content. Food Hydrocolloids.

[cit77] Lecorre D., Bras J., Dufresne A. (2012). Influence of native starch's properties on starch nanocrystals thermal properties. Carbohydr. Polym..

[cit78] Lecorre D., Bras J., Dufresne A. (2012). Influence of native starch's properties on starch nanocrystals thermal properties. Carbohydr. Polym..

[cit79] Rehman S., Ullah R., Butt A. M., Gohar N. D. (2009). Strategies of making TiO2 and ZnO visible light active. J. Hazard. Mater..

[cit80] Caló E., Khutoryanskiy V. V. (2015). Biomedical applications of hydrogels: A review of patents and commercial products. Eur. Polym. J..

[cit81] Caló E., Khutoryanskiy V. V. (2015). Biomedical applications of hydrogels: A review of patents and commercial products. Eur. Polym. J..

[cit82] Lu X., Zhang H., Ni Y., Zhang Q., Chen J. (2008). Porous nanosheet-based ZnO microspheres for the construction of direct electrochemical biosensors. Biosens. Bioelectron..

[cit83] Lu X., Zhang H., Ni Y., Zhang Q., Chen J. (2008). Porous nanosheet-based ZnO microspheres for the construction of direct electrochemical biosensors. Biosens. Bioelectron..

[cit84] Salehi R., Arami M., Mahmoodi N. M., Bahrami H., Khorramfar S. (2010). Novel biocompatible composite (Chitosan–zinc oxide nanoparticle): preparation, characterization and dye adsorption properties. Colloids Surf., B.

[cit85] Joye I. J., Davidov-Pardo G., McClements D. J. (2014). Nanotechnology for increased micronutrient bioavailability. Trends Food Sci. Technol..

[cit86] Hu W., Yu X. (2012). Encapsulation of bio-based PCM with coaxial electrospun ultrafine fibers. RSC Adv..

[cit87] Hu W., Yu X. (2012). Encapsulation of bio-based PCM with coaxial electrospun ultrafine fibers. RSC Adv..

[cit88] Liao W., Badri W., Dumas E., Ghnimi S., Elaissari A., Saurel R., Gharsallaoui A. (2021). Nanoencapsulation of Essential Oils as Natural Food Antimicrobial Agents: An Overview. Appl. Sci..

[cit89] Liao W., Badri W., Dumas E., Ghnimi S., Elaissari A., Saurel R., Gharsallaoui A. (2021). Nanoencapsulation of Essential Oils as Natural Food Antimicrobial Agents: An Overview. Appl. Sci..

[cit90] Buenger D., Topuz F., Groll J. (2012). Hydrogels in sensing applications. Prog. Polym. Sci..

[cit91] Dragan E. S., Apopei D. F. (2013). Multiresponsive macroporous semi-IPN composite hydrogels based on native or anionically modified potato starch. Carbohydr. Polym..

[cit92] Dragan E. S., Apopei D. F. (2013). Multiresponsive macroporous semi-IPN composite hydrogels based
on native or anionically modified potato starch. Carbohydr. Polym..

[cit93] Zheng H., Zuo B. (2021). Functional silk fibroin hydrogels: preparation, properties and applications. J. Mater. Chem. B.

[cit94] Kausar A. (2021). Progress in green nanocomposites for high-performance applications. Mater. Res. Innovations.

[cit95] Tang X., Alavi S., Herald T. J. (2008). Barrier and Mechanical Properties of Starch-Clay Nanocomposite Films. Cereal Chem..

[cit96] Mishra R. K., Ha S. K., Verma K., Tiwari S. K. (2018). Recent progress in selected bio-nanomaterials and their engineering applications: an overview. J. Sci.: Adv. Mater. Devices.

[cit97] Rossi M., Cubadda F., Dini L., Terranova M. L., Aureli F., Sorbo A., Passeri D. (2014). Scientific basis of nanotechnology, implications for the food sector and future trends. Trends Food Sci. Technol..

[cit98] Ochi S. (2008). Mechanical properties of kenaf fibers and kenaf/PLA composites. Mech. Mater..

[cit99] Uddin A. J., Araki J., Fujie M., Sembo S., Gotoh Y. (2012). Interfacial interaction and mechanical properties of chitin whisker–poly(vinyl alcohol) gel-spun nanocomposite fibers. Polym. Int..

[cit100] Uddin A. J., Araki J., Fujie M., Sembo S., Gotoh Y. (2012). Interfacial interaction and mechanical properties of chitin whisker–poly(vinyl alcohol) gel-spun nanocomposite fibers. Polym. Int..

